# Time Variable Models of Severe Hemorrhagic Shock in Rats

**DOI:** 10.3390/life15040522

**Published:** 2025-03-22

**Authors:** Matthew B. Barajas, Takuro Oyama, Miriam J. K. Walter, Masakazu Shiota, Zhu Li, Matthias L. Riess

**Affiliations:** 1 Department of Anesthesiology, Vanderbilt University Medical Center, Nashville, TN 37232, USA; taku0619uro12@gmail.com (T.O.); zhu.li@vumc.org (Z.L.); matthias.riess@vanderbilt.edu (M.L.R.); 2 Department of Anesthesiology, Tennessee Valley Healthcare System, Veterans Affairs Medical Center, Nashville, TN 37212, USA; 3 Department of Anesthesiology, Tokushima University, Tokushima 770-8503, Japan; 4 Department of Anesthesiology, Universitätsmedizin Greifswald, 17475 Greifswald, Germany; miriam_walter@t-online.de; 5 Department of Molecular Physiology and Biophysics, Vanderbilt University School of Medicine, Nashville, TN 37232, USA; shiotamasakazu@gmail.com; 6 Department of Pharmacology, Vanderbilt University, Nashville, TN 37232, USA

**Keywords:** hemorrhage, resuscitation, severity, trauma, volume management, golden hour

## Abstract

Background: Classical teaching dictates that damage control resuscitation is ideally implemented within the first or ‘golden’ hour after trauma-related hemorrhage. Given the heterogeneity of trauma, varied models must be utilized to guide ongoing investigation. We sought to determine exactly what happens during the ‘golden hour’ by varying hemorrhage and down times and mimicking venous or arterial bleeding while varying oxygen therapy, a readily available pre-hospital intervention, on survival in a small-animal rodent model. Methods: Rats were bled by 40% of their blood volume over 30 or 60 min, with varied ‘down-times’ of 30, 45, or 60 min. F_i_O_2_ was administered at 21% or 40%, mimicking nasal cannula. Multiple linear regression was performed between the independent variables and each measured outcome. Sub-group analyses were stratified by survival. Results: There was no statistically significant variation in end-organ insult (lactate), cardiac functioning (cardiac output or left ventricle fractional area of change), mean arterial pressure at end experiment, survival, or survival times among the groups. Conclusions: This study adds to the data against an all-encompassing golden hour, as even a rapid hemorrhage with long down time did not decrease survival. Furthermore, we add to the body of literature in this field by examining cardiac markers of injury with transthoracic echocardiography.

## 1. Introduction

There is a long-standing concept in traumatic hemorrhage and shock that treatment should be initiated within the ‘golden hour’ to improve patient outcomes [1]. Debate wages on whether there are enough meaningful data to support the concept of this crucial resuscitation window [2,3]. While delaying treatment is never the first choice of providers, there are situations where time management plays a critical role in resuscitation. Mass casualty events where there are strains on resources, e.g., remote location trauma, scene safety issues, and battlefield scenarios, may delay treatment. By its nature, trauma is heterogeneous; thus, treatment will also vary. Severe injury, such as aortic transection, may necessitate resuscitation well before the ‘golden hour’, whereas primarily distal orthopedic injury, tibia and fibula, or radius and ulna fractures, may allow for a more relaxed pace of care. One retrospective study of military casualties in Afghanistan found that pre-hospital resources (blood transfusion capability), location of dominant trauma, blunt vs. penetrating trauma, and transport times all factored into survival [4]. In penetrating trauma, the vascularity of injured organs and damage to major venous vs. arterial structures could affect the rate of hemorrhage.

The management of hemorrhagic shock is two-fold. Providers must stop the ongoing source of bleeding while also delivering volume resuscitation. Current resuscitation guidelines emphasize the minimization of pre-hospital crystalloid use, as it is associated with mortality, multi-organ failure, and dilutional coagulopathy [5]. However, as discussed above, pre-hospital constraints and transport times may make blood-predominant early resuscitation infeasible. In one study of patients requiring transport by EMS helicopter, an optimal prehospital crystalloid volume of 250–1250 mL was associated with decreased mortality [6]. In blood-constrained areas, such as Cameroon, the delay in receiving blood offset the benefits when compared to crystalloid-based resuscitation [7].

Several models of hemorrhagic shock exist in pre-clinical animal studies [8]. Rats remain one of the most popular small animal models due to the ease of use and cost, as well as the parallel in immune and inflammatory responses to trauma and hemorrhagic shock. However, there are distinct differences in hemodynamic responses between rats and humans [8]. Both pressure- and volume-centered models have been used in rats. In fixed pressure models, a goal MAP range is established, often between 30 and 45 mmHg, and volume is either removed or redelivered to meet this goal over a chosen period of insult time. In fixed volume models, 30–50% of the animal’s blood volume is removed over a fixed period. Additionally, models who are awake or under light anesthesia have been used to limit anesthetic preconditioning and hypotension associated with anesthesia [9]. Uncontrolled hemorrhage can also be performed using tail amputation [10]. The multitude of current models may lead to confusion and decrease the external validity of the data to date.

As vascular beds contract in response to hypovolemia, reductions in microcirculatory flow lead to the cellular oxygen deprivation of tissue beds [11]. Supplemental oxygen may partially reverse tissue hypoxia at low hemorrhage levels. However, in severe hemorrhage or cases with severe hyperoxemia, lower tissue partial pressure of oxygen may paradoxically occur [12]. Reactive oxygen species may increase during reperfusion and worsen cellular injury [13]. While some studies demonstrate increased end organ ischemia with supplemental oxygen, others demonstrate improved survival [12,14,15].

Given the heterogeneity of the disease process, volume and rate of hemorrhage, and the treatment, volume, and resuscitation product, various models must be investigated to better understand managing this acute insult. We sought to determine the effects of hemorrhage rates (fixed volume over varied times), hemorrhagic shock ‘down’ times, and oxygen therapy, a readily available pre-hospital therapy, on survival in a small-animal rodent model.

## 2. Materials and Methods

Institutional Animal Care and Use Committee approval was obtained for all study procedures (Protocol M1800029-01, 6 January 2022, Vanderbilt University Medical Center, Nashville, TN, USA). Subjects were cared for in accordance with the Vanderbilt University Medical Center Division of Animal Care guidelines, including continuously available food and water, 12 h light cycles, social housing and enrichment. Experiments were conducted according to guidelines in the Animal Research: Reporting of In Vivo Experiments.

Forty adult male Sprague Dawley rats (Inotiv Inc., Indianapolis, IN, USA) with experimental weights of 479 ± 60 g were used. The subjects were initially anesthetized with isoflurane 5% via room air at high flow for 5 min into their standard housing cage. After induction, animals were weighed, shaved, and intubated with a 14-gauge (G) catheter (BD Insyte Autogard, Becton, Dickinson and Co., Ltd., Franklin Lakes, NJ, USA) using video laryngoscopy [16]. Once intubated, the animals were mechanically ventilated in a volume-controlled manner, 8 mL kg^−1^ tidal volume, with continuous isoflurane inhalation at 1.5% inspired fraction. Fraction of inspired oxygen (F_i_O_2_) was adjusted per a manual air/oxygen mixer. Normothermia was maintained using a heating mat (T/Pump Professional, Stryker Corp., Kalamazoo, MI, USA) to achieve the target temperature of 36.5 to 37.5 °C as measured via rectal thermometer.

Once anesthetized and positioned supine, surgical cutdown was performed to access and cannulate vessels [13]. Polyethylene 25 tubing (Instech Laboratories Inc., Plymouth Meeting, PA, USA) connected to a 25 G blunt needle (Air-Tite Products Co., Inc., Virginia Beach, VA, USA) was advanced into vessels for access, and lines were flushed with normosol-R (Phizer Inc., New York, NY, USA). Arterial pressure from the femoral artery was measured using TruWave pressure transducers (Edwards Lifesciences Corp, Irvine, CA, USA). The femoral vein was used to hemorrhage and to deliver blood and crystalloid volume. Cautery (HIT1, Bovie High-temp cautery kit, Symmetry Surgical Inc., Antioch, TN, USA) was used to ensure dry surgical beds and, thus, no continued volume loss that was not from experimental blood withdraw.

Hemorrhage and resuscitation were performed using a time and volume variable syringe pump (Braintree Scientific, Inc., Braintree, MA, USA). Blood volume (BV) was determined by body weight (BW) utilizing the following equation: BV = 0.06 × BW+ 0.77 [17]. Forty percent of the blood volume was ‘hemorrhaged’ by being withdrawn into a heparinized syringe via the femoral venous cannula. Later in the resuscitation phase, the blood was redelivered over 15 min. Between these phases, blood was held in the heparinized syringe at room temperature and not exposed to air. The time over which blood was withdrawn, hemorrhage time, was varied to 30 or 60 min, and time between end of hemorrhage and the beginning of resuscitation, down time, varied to 30, 45, or 60 min in this experimental protocol. F_i_O_2_ was also varied in the experimental protocol as previously described, to room-air 21%, or 40%, mimicking maximal oxygen delivery via a nasal cannula. Therefore, 12 total combinations of hemorrhage time, down time, and F_i_O_2_ were evaluated for outcomes in this experiment. Resuscitation and monitoring time was fixed at two hours maximum.

Outcomes studied included survival, time to death, lactate, mean arterial pressure (MAP), fractional area of change (FAC) of the left ventricle, and calculated cardiac output (CO). Lactate was measured using an arterial blood sample in a point of care device (Nova Biomedical Corp., Waltham, MA, USA). Vital signs were recorded using Powerlab Series 16/30 in LabChart (Version 8.1.13, AD Instruments North America Inc., Colorado Springs, CO, USA). Transthoracic echocardiography (Philips Affiniti 50, Philips Ultrasound Inc., PA, USA) was performed to determine left ventricular function, and CO was calculated using the main pulmonary artery diameter and flow as measured by velocity time integral via pulse wave Doppler.

Statistical analysis was performed using SigmaStat (ver 3.5, Systat Software Inc., San Jose, CA, USA). First, multiple linear regression was performed between the independent variables (hemorrhage time, down time, and F_i_O_2_) and baseline values across groups for each study variable. This was conducted due to the small sample size in each group, to ensure no baseline bias or variability across groups. Next, multiple linear regression was performed between the independent variable and each measured outcome. Sub-group analysis was performed for those who did or did not survive to the end experiment. Significance was set at *p* ≤ 0.05, two-tailed.

## 3. Results

The initial analysis performed was to ensure that no basal inequalities between groups were associated with our independent variables: hemorrhage time, down time, and F_i_O_2_. The total N value for each group can be seen in Table 1. Animal size is correlated with vascular size, and cannulation can be significantly easier in larger animals. If easier, there may be less blood loss which could affect outcomes. No correlation was present on regression analysis, *p* = 0.55, 0.41 and 0.41 for hemorrhage time, down time and F_i_O_2_, respectively. Baseline lactate can be used to assess pre-hemorrhage state of end-organ ischemia. There should be no basal ischemic insult and no significant correlation of baseline lactate with the independent variables, *p* = 0.15, 0.47 and 0.3, respectively. There was no significant difference in baseline MAP among groups, with no correlation with independent variables, *p* = 0.9, 0.24 and 0.11, respectively. There was no correlation of baseline CO with independent variables, *p* = 0.17, 0.7 and 0.63, respectively. There was, however, a significant correlation between FAC and F_i_O_2_ where increased F_i_O_2_ was correlated with a decreased FAC at baseline, *p* = 0.045. There was no correlation between FAC and hemorrhage or down times, *p* = 0.37 and 0.56, respectively.

**Figure 1:**
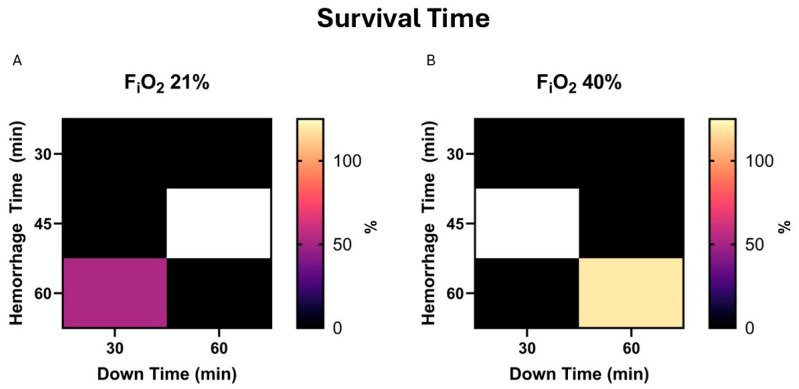
(**A**). Survival time is demonstrated in a heat map for 21% F_i_O_2_. Here, survival time is calculated as the time remaining in the experiment. Zero (darker) indicates survival to the end of the experiment, while greater values (brighter) indicate early death and a more severe model. (**B**). The same map demonstrates values in the presence of 40% F_i_O_2_.

Subsequent analysis evaluated relationships between study variables and independent variables. Unexpectedly, there was no significant correlation between survival and hemorrhage time, downtime or F_i_O_2_, *p* = 0.55, 0.46, and 0.97, respectively. Therefore, the animal hemorrhaging faster or remaining in a state of shock for longer did not affect outcomes in our study. This was evaluated with logistic regression, using the dichotomous alive or dead method at the end of the two-hour resuscitation period. Figure 1 expands upon this analysis, evaluating the time remaining in the experiment. Thus, a value of 0 indicates survival. This retrograde measurement was used, as the time from the start of experiments to the resuscitation period varied based on hemorrhage and down times, and, thus, may have skewed antegrade based results. There was no correlation between independent variables and time of death based on multiple linear regressions, *p* = 0.5, 0.34, and 0.79 for hemorrhage time, down time, and F_i_O_2_.

**Figure 2:**
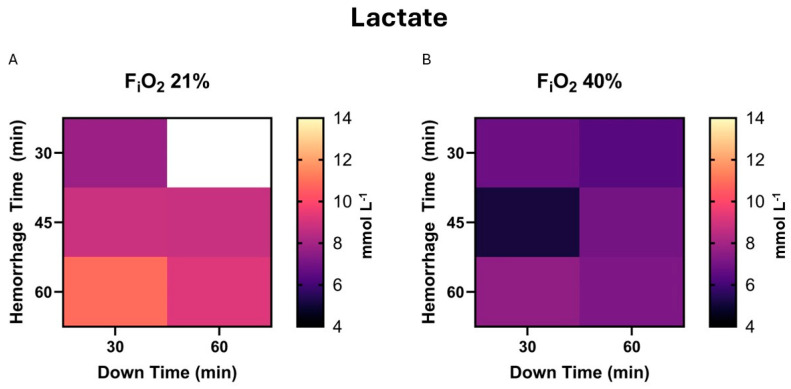
(**A**). The highest measured lactate during the experiment, or peak lactate, is demonstrated in heat map form for those exposed to 21% F_i_O_2_. This includes survival and non-survival animals. (**B**). The same map demonstrates values in the presence of 40% F_i_O_2_.

As none of the independent variables was associated with survival, we sought to test if there was a correlation between additional variables and survival. Baseline lactate did not affect survival, *p* = 0.959, nor survival time, *p* = 0.16. In a subgroup analysis of those who did not survive, a higher baseline lactate was correlated with more prolonged survival, *p* = 0.025. In the subgroup analysis of survivors, those with a lower hemorrhage time, had a lower lactate at the end experiment, *p* = 0.67. Down time and F_i_O_2_ were not significantly correlated with lactate, *p* = 0.52 and 0.11, respectively. A heat map demonstrating peak lactate during the experiment is presented in Figure 2.

**Figure 3:**
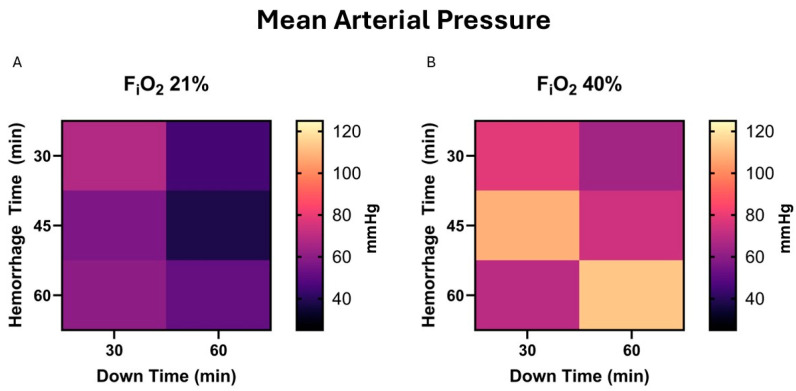
(**A**) Mean arterial pressure (MAP) at end-experiment in those who survived is demonstrated in a heat map for 21% F_i_O_2_. This does not include animals who did not survive, e.g., the MAP was <30 mmHg 2 h after beginning resuscitation. (**B**). The same map demonstrates values in the presence of 40% F_i_O_2_.

There was no significant correlation between MAP and survival, *p* = 0.52, nor survival time, *p* = 0.65. There was no correlation between independent variables and the MAP at the end experiment, *p* = 0.47, 0.16, and 0.51 for hemorrhage time, down time, and F_i_O_2_; Figure 3.

**Figure 4:**
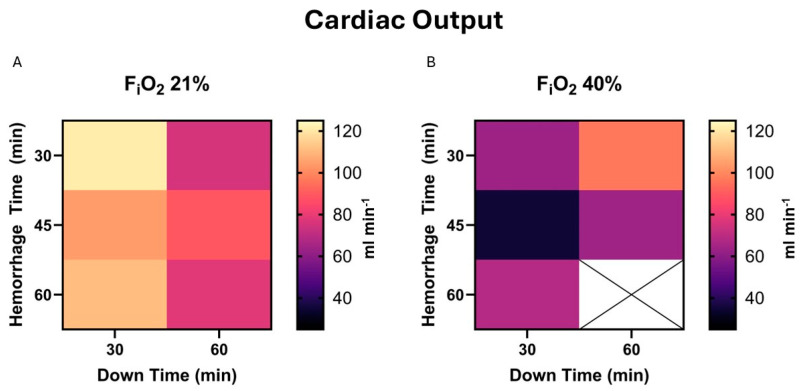
(**A**). Cardiac output (CO) at end-experiment in those who survived is demonstrated in a heat map for 21% F_i_O_2_. This does not include animals who did not survive, e.g., the MAP was <30 mmHg 2 h after beginning resuscitation. CO is calculated based on transthoracic echocardiography assessment of flow through the main pulmonary artery. (**B**). The same map demonstrates values in the presence of 40% F_i_O_2_. There was insufficient data to fill the 60 min hemorrhage and 60 min down times square.

CO is the most important marker of cardiac insult and resultant function. There was no correlation between independent variables and the CO at end-experiment, *p* = 0.29, 0.41, and 0.25 for hemorrhage time, down time, and F_i_O_2_; Figure 4.

**Figure 5:**
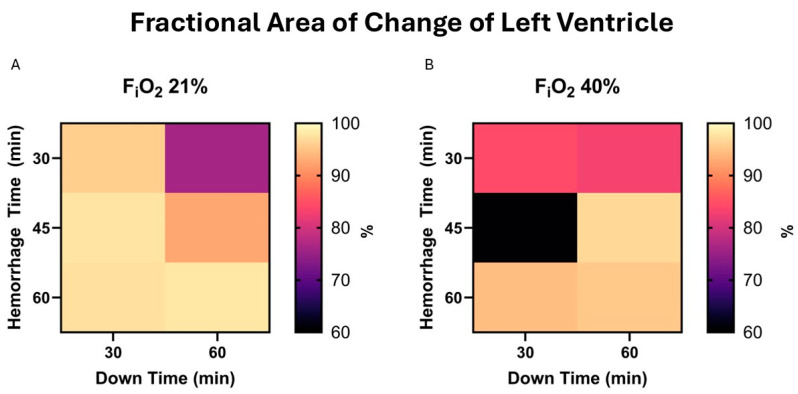
(**A**). Fractional area of change (FAC) at end-experiment in those who survived is demonstrated in a heat map for 21% F_i_O_2_. This does not include animals who did not survive, e.g., the MAP was <30 mmHg 2 h after beginning resuscitation. (**B**). The same map demonstrates values in the presence of 40% F_i_O_2_.

Fractional area change is the 2-dimensional equivalent to the commonly assessed left ventricular ejection fraction. FAC aims to assess left ventricular function, while CO assesses both ventricles combined. There was no significant correlation between independent variables and FAC at end-experiment in survivors, *p* = 0.56, 0.6, and 0.67 for hemorrhage time, down time, and F_i_O_2_; Figure 5.

## 4. Discussion

The use of various models of hemorrhage (time-dependent, volume-dependent, MAP targeted, or a combination, and across varied ‘down times’) has led to varying results regarding management of hemorrhagic shock. However, in our current study, we demonstrate that physiologic end-points have similar outcomes despite a range of hemorrhage rates and down times and may not be as disparate as thought. Our study had an overall survival rate of 65%, demonstrating a severe but survivable model. We believe this insult level appropriately combines external validity while maintaining achievability in the pre-clinical period. A notable limitation is that we did not investigate an untreated shock model as a baseline comparator of survival rate, as this would almost assuredly lead to 100% mortality.

One method not studied here is a fixed volume/variable hemorrhage rate model. Some have proposed this as a more physiologic model, as the MAP drops due to hemorrhage, further hemorrhage should theoretically slow down as perfusion pressure to the injured tissue decreases. Limited studies in pigs have shown that there is an increased physiologic insult using this technique, but this has not been studied in rats [18]. It is unclear why this insult would be stronger, as this mechanism seems to limit blood loss, and is the concept behind purposefully hypotensive resuscitation in trauma settings.

**Figure 6:**
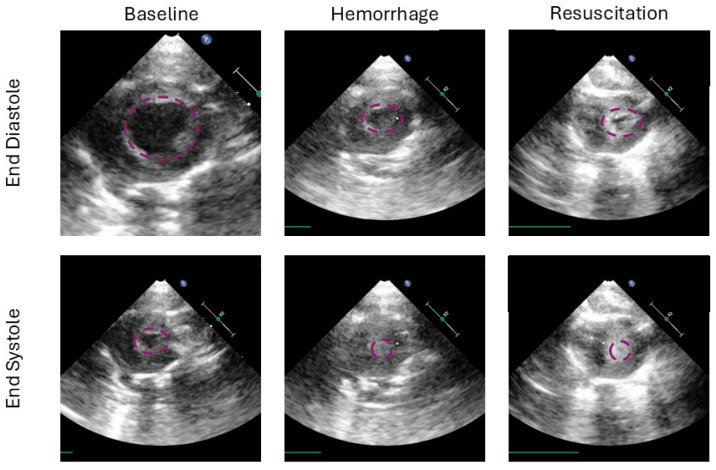
This figure demonstrates the left ventricle in end diastole (**top row**) and end systole (**bottom row**) at several time points in the study (from left to right: baseline, at the end of hemorrhage and the end of the experiment after resuscitation). The baseline columns show a robust end diastolic volume, and a residual cavity in systole. Ventricular area is highlighted in purple for each image. After hemorrhage, the ventricle is underfilled and the systolic cavity is not present, representing 100% ejection. In the resuscitation column we see residual underfilling, however, semblance of a cavity can be seen, suggesting either cardiac injury, ongoing reduction in preload, or a combination.

The strength of this study may also be its limitation. Here, we evaluate many groups to tease apart how the hemorrhage and down time components contribute to the ‘golden hour’. To achieve this required a small N per group, 3–4. While our groups were evenly matched at baseline per our analysis of basal characteristics, there was a significant variation in survival and time to death within groups of the same treatment. This could have been improved with more repetitions, which was not feasible in the current study. Given the complex structure of this study, no traditional sample size calculations were performed a priori. Resultant to this, several calculations were left with a power below 0.8, therefore, type II error in the case of a negative result cannot be excluded. Alternatively, the limitation of two hours of post resuscitation time may have limited a survival signal that could have appeared later on. However, there were high homogeneity levels in lactate and MAP at end-experiment within groups, which are commonly assessed markers in trauma and shock models. Furthermore, this study added the first assessment of cardiac end-organ ischemia assessed with transthoracic echocardiography in a severe hemorrhagic shock model, Figure 6. By assessing left ventricle function and cardiac output in these models, future studies can evaluate systemic vascular resistance after resuscitation, which may be altered from post-ischemic vasodilation and any myocardial damage from hypoperfusion. What we have taken away from the left ventricular evaluation, is that after resuscitation the end-diastolic diameter did not return to baseline, and the FAC remained elevated. This is indicative of continued ‘hypovolemia’, which in our tightly controlled closed system study, means that the ischemia–reperfusion after hemorrhagic shock results in a vasodilatory response or to third spacing and tissue swelling to a significant degree. We believe the former to be more significant given the time frame of this study. This has repercussions for trauma resuscitation, meaning that normovolemia or normalizing left ventricular end diastolic diameter may not be reflective of the patient’s true baseline volume status. Lactatemia and acidosis after reperfusion should be evaluated in the overall picture and some vasoconstrictive agents may be appropriate if hypervolemia is to be avoided. Notable hypervolemia in the perioperative space is well studied and linked with poor outcomes [19]. That controlled setting may be less severe than what we see in this study.

Given the severity and variability of hemorrhagic insult, this study may have lacked sufficient power to detect a difference in outcomes with low-grade oxygen therapy. However, there may be a trend towards increased MAP and lower lactate at end-experiment in those treated with supplemental oxygen therapy. This did not correlate with a survival trend, but that may be due to the variability in hemorrhagic insults in this study. Overall, 100% oxygen therapy at both normobaric and hyperbaric pressures has been shown to improve blood pressure and increase survival rates [9,15]. Currently, it is unclear whether the effect of oxygen therapy on blood pressure is a function of cardiac output or systemic vascular resistance or a combination of both. The work outlined in this study may be a basis for further investigation. However, contrary evidence that oxygen therapy decreases tissue perfusion also exists [12]. Long-term outcomes require further investigation.

## 5. Conclusions

Trauma remains the most common etiology of death in the first four decades of life [20]. A large portion of hemorrhagic shock is a treatable disease as long as it is recognized and the resources for treatment are available. One barrier to treatment is time, and the classic teaching of resuscitation within the ‘golden hour’ lacks strong evidence. Here, we aimed to determine exactly what happens during the ‘golden hour’ based on hemorrhage times, and down times, mimicking venous or arterial bleeding with some field aid while transport to a care facility is performed. Additionally, we investigated the role of low-dose supplemental oxygen therapy as a treatment for tissue hypoxemia. We found a significant level of heterogeneity in hemodynamics across hemorrhage and down times, with similar end-organ insults and survival outcomes. This adds to the data against an all-encompassing golden hour. Furthermore, we add to the body of literature in this field by examining cardiac markers of ischemia and reperfusion injury with transthoracic echocardiography. Further studies should focus on the effects of oxygen therapy both on vascular tone and end-organ perfusion.

## Figures and Tables

**Table 1:** Table depicting total N of each subgroup.

Total N Per Group					
		21% F_i_O_2_		40% F_i_O_2_	
		**Hemorrhage Time (min)**			
		**30**	**60**	**30**	**60**
**Down Time (min)**	**30**	3	3	3	2
	**45**	3	4	3	3
	**60**	3	4	4	4

## Data Availability

All data are available upon reasonable request and in accordance with funding guidelines.

## Abbreviations

The following abbreviations are used in this manuscript:

G	Gauge
F_i_O_2_	Fraction of inspired oxygen
BV	Blood volume
BW	Body Weight
MAP	Mean arterial pressure
FAC	Fractional area of change
CO	Cardiac output
